# The Pregnancy Outcome of Mosaic Embryo Transfer: A Prospective Multicenter Study and Meta-Analysis

**DOI:** 10.3390/genes11090973

**Published:** 2020-08-21

**Authors:** Ying Xin Zhang, Jang Jih Chen, Sunanta Nabu, Queenie Sum Yee Yeung, Ying Li, Jia Hui Tan, Wanwisa Suksalak, Sujin Chanchamroen, Wiwat Quangkananurug, Pak Seng Wong, Jacqueline Pui Wah Chung, Kwong Wai Choy

**Affiliations:** 1 Department of Obstetrics and Gynaecology, The Chinese University of Hong Kong, Hong Kong 999077, China; elynnzhang@link.cuhk.edu.hk (Y.X.Z.); queenieyeung@cuhk.edu.hk (Q.S.Y.Y.); liying_erin@link.cuhk.edu.hk (Y.L.); jacquelinechung@cuhk.edu.hk (J.P.W.C.); 2 Sunfert International Fertility Centre Sdn. Bhd, Kuala Lumpur 59200, Malaysia; aaron.chen@sunfert.com (J.J.C.); jia.hui.tan@sunfert.com (J.H.T.); pak.seng.wong@sunfert.com (P.S.W.); 3 Safe Fertility Center Co. Ltd., Bangkok 10330, Thailand; nanta.buna@gmail.com (S.N.); aorpoul@hotmail.com (W.S.); sujin.ch@safefertilitycenter.com (S.C.); dr.wiwat@safefertilitycenter.com (W.Q.)

**Keywords:** preimplantation genetic testing for aneuploidies (PGT-A), mosaic embryo transfer (MET), meta-analysis, multi-center, prospective

## Abstract

Chromosomal mosaicism is at high occurrence in early developmental-stage embryos, but much lower in those at prenatal stage. Recent studies provided evidence on the viability of mosaic embryos by reporting pregnancy outcomes. Expanded research is warranted to evaluate its clinical significance. This is a multi-center prospective cohort study on 137 mosaic, 476 euploid and 835 non-preimplantation genetic testing (non-PGT) embryos from three in vitro fertilization (IVF) providers of three countries in Asia, applying the same preimplantation genetic testing for aneuploidies (PGT-A) reporting criteria. Mosaic embryo transfers (METs) resulted in a significantly lower clinical pregnancy rate (40.1% versus 59.0% versus 48.4%), lower ongoing/live birth rate (27.1% versus 47.0% versus 35.1%) and higher miscarriage rate (33.3% versus 20.5% versus 27.4%) than euploid and non-PGT transfers, respectively. Pregnancy losses after METs were different between embryos carrying numerical and segmental chromosomal abnormalities (*p* = 0.04). Our meta-analysis concluded that METs gave rise to pregnancies but were associated with a reduced ongoing/live birth rate and a higher miscarriage rate. All 37 MET live births were confirmed viable, among which 8 completed prenatal genetic testing with normal results. Longitudinal investigation on one MET pregnancy evidenced the aneuploidy depletion hypothesis. This is the first multi-center prospective study reporting a full MET pregnancy outcome with complementary information from prenatal genetic testing as compared to euploid and non-PGT cohorts.

## 1. Introduction

Mosaicism has been reported in cleavage and blastocyst stage embryo biopsies. Chromosomal mis-segregation during either meiosis or mitosis results in aneuploidy. Age has been well characterized as the cause of aberrant chromosomal separation in gametogenesis, which leads to constitutional aneuploidy. Mitotic errors in early embryonic development have been characterized as the mechanism of chromosomal mosaicism, producing two or more cell lines within one embryo or organism. Current preimplantation genetic testing for aneuploidies (PGT-A) has a common resolution at 5 Mb to 10 Mb by sequencing and a reproducible detection limit of the mosaic level at 20% or 30% [1,2,3]. The current utility of PGT-A varies in platforms and reporting criteria and, as a result, controversies have arisen regarding the number of utilizable embryos per cycle. In an extensive cohort study from pregnancies with confined placental mosaicism, karyotyping on chorionic villi sampling (CVS) detected 1.3% cases as confined placental mosaicism, and 0.2% as true fetal mosaicism confirmed by amniocentesis [4]. On the other perspective, the incidence of embryonic mosaicism reported in PGT-A was 3% to 40% [5,6,7]. Besides, while selecting euploid embryos for transfer can achieve a 50% ongoing pregnancy rate in good-prognosis patients, this is not significantly different from transferring embryos selected by morphological grading alone [7]. This phenomenon implies that a proportion of karyotypically normal pregnancies may have originated from mosaic embryos.

Greco et al. [8] first reported healthy live births from mosaic monosomy embryos at mosaic levels ranging from 35% to 50% and affecting different chromosomes. Soon published mouse model supported this finding as a consequence of aneuploid cell depletion [8] in addition to trisomy rescue during embryo development [9,10]. Four previous studies illustrated successful pregnancies by transferring embryos carrying different levels of mosaicism [2,11,12,13], a number of mosaic chromosomes [2,12,13,14], a chromosomal copy number change [12] and mosaic segmental aneuploidies [2,15]. The Preimplantation Genetic Diagnosis International Society (PGDIS) announced its position statement in 2016 and later updated this in 2019 [16], providing guidelines for mosaic embryo transfers (METs).

Since several studies have confirmed the lowered implantation potential of mosaic embryos, the major concern of METs remains to be the factor(s) that may affect live birth rate, as well as the antenatal and postnatal health of babies derived from mosaic embryos. Therefore, we conducted this multi-center prospective cohort study with the aim of characterizing factors that affect live birth rate and obtaining additional information from prenatal and/or postnatal genetic follow-ups of MET pregnancies.

## 2. Materials and Methods

### 2.1. Ethical Approval

This study was approved by the Joint Chinese University of Hong Kong-New Territories East Cluster Clinical Research Ethics Committee (CREC-2010.432 and CREC-2007.059). Patient gave their written informed consent before inclusion.

### 2.2. Study Design

This study was conducted at three in vitro fertilization- preimplantation genetic testing (IVF-PGT) centers in Hong Kong (CUHK-PWH, the Chinese University of Hong Kong-Prince of Wales Hospital), Malaysia (Sunfert International Fertility Center) and Thailand (Safe Fertility Center). The study period spanned from 2016 to 2018 and recruited all IVF and Intracytoplasmic sperm injection (ICSI) cycles with embryo transfers. The viability of mosaic embryos was studied by reviewing the pregnancy outcome and making comparisons with an age-matched control group. A total of 137 mosaic embryos transferred in 133 frozen-embryo transfer (FET) cycles (including 129 single- and 4 double-embryo transfers) and 476 euploid embryo transferred in 447 FET cycles (including 418 single- and 29 double-embryo transfers) from 3 centers were included in the MET group and euploid control group, respectively. In addition, 835 embryos from 777 non-PGT cycles were included as a non-PGT control group (Table 1).

Prior to the detection and reporting of mosaicism, cell line mixes by gradual proportions of mosaicism (0%, 16.7%, 33.3%, 50%, 66.7%, 83.3%, 100%) were tested for validation of both chromosomal microarray analysis (Agilent Technologies, United States) and next-generation sequencing (NGS) by VeriSeq (Illumina, Inc., San Diego, CA, USA) platforms. This study was designed in three parts. The first part was a multicenter prospective cohort study of pregnancy outcome of METs. Two centers used VeriSeq platform while one center used chromosomal microarray analysis (CMA) for PGT-A. With the multi-center data, a meta-analysis was performed to summarize the overall risk ratio of pregnancy outcome by the MET approach. Thirdly, one MET patient was longitudinally followed up from preimplantation to the prenatal and postnatal stages. Biopsies from multiple placental sites were tested by CMA.

### 2.3. Embryo Culture and Biopsy

Two centers cultured embryos in G-TL medium (Vitrolife, Gothenburg, Sweden) and one in G1/G2 sequential media (Vitrolife, Sweden) supplemented with 10% synthetic serum substitute (SSS, Irvine Scientific, Santa Ana, CA, USA). Assisted hatching was performed using non-contact laser (Research Instrument, Falmouth, UK). Laser-assisted isolation of trophectoderm (TE) was performed on day 5 or day 6 of embryo development if a blastocyst had discernable inner cell mass and TE. Morphological grading of blastocysts was assessed according to Schoolcraft and Gardner [17]. Blastocyst quality was grouped and classified using the simplified Society for Assisted Reproductive Technology (SART) embryo scoring system [18].

### 2.4. PGT-A

Chromosomal microarray analysis by Agilent 8 × 60 K microarray chip and the NGS by VeriSeq PGS kit were conducted according to the manufacturer’s protocols. Data analysis was performed with Cytogenomics 2.7.8.0 software (Agilent) for CMA and BlueFuse Multi for NGS (Illumina, Inc.). Both pipelines used human genome build 19 (hg19/GRCh37) as a reference genome.

The NGS-based PGT-A method is characterized by having high detection resolution for segmental aneuploidies [19,20]. Based on in-house validation, the resolution of segmental aneuploidy was set as 10 Mb for both CMA and NGS PGT-A platforms [21,22]. The level of mosaicism was calculated by the copy number ratio (Equation (1)) and reported with a range of 20% to 80% mosaic level.

Level of mosaicism (%) = |(copy ratio − 1)/0.5 × 100|(1)

### 2.5. Genetic Counselling

The option of mosaic embryo transfer would only be provided to patients when they had no euploid embryo. Genetic counseling was provided by clinical geneticists for the reasons, options, potential outcomes and prenatal management of transferring mosaic embryos [16,23,24].

Patients were highly recommended to perform prenatal diagnosis during genetic counselling. Prenatal genetic testing results were traced on pregnancies that were sustained beyond the first trimester. Non-invasive prenatal testing (NIPT) as the prenatal screening approach was traced and recorded by all three centers in this study. Genetic testing on amniotic fluid or chorionic villi by either karyotyping or CMA was performed in the case of prenatal genetic diagnosis. Karyotyping or CMA was performed on the product of gestation (POG) on patients’ request.

### 2.6. Outcome Analysis

Pregnancy outcome was traced and calculated at four stages according to nomenclatures [25,26]: (i) Implantation and clinical pregnancy was defined as a gestational sac seen by ultrasound. Implantation rate (IR) was the number of embryos implanted among number of embryos transferred. Clinical pregnancy rate (CPR) represented the number of clinical pregnancies among the total number of ETs. (ii) Ongoing pregnancy was a pregnancy that was sustained beyond clinical pregnancy but had not yet delivered at the point of follow-up. Ongoing pregnancy rate (OPR) represented the percentage of ongoing pregnancies out of the total number of ETs. (iii) Live birth was viable delivery, and live birth rate (LBR) was the proportion of live-born deliveries over the total number of embryo transfers. As 111 embryos from 105 euploid embryo transfers had only reported ongoing pregnancies but not deliveries, ongoing pregnancies and live births were combined for analysis in this study. (iv) Miscarriage was defined as clinical pregnancies that did not result in live birth. Miscarriage rate was calculated as the number of miscarriage pregnancies among the total number of clinical pregnancies.

### 2.7. Placenta Biopsy

One MET pregnancy had full-term delivery with the whole placenta donated. Placenta biopsy was performed on 16 sites: 8/16 from the maternal side and 8/16 from the fetal side. Biopsy sites were randomly selected from different cotyledons that were naked-eye identifiable. Chromosomal microarray analysis (CMA; 8 × 160 k, Agilent) was performed on all 16 sites. As the sex of the fetus is known to be a male, male control was selected as a reference for CMA.

### 2.8. Meta-Analysis

The present meta-analysis was performed following the preferred reporting item for systematic reviews and meta-analysis (PRISMA) statement [27]. The electronic database Pubmed was searched up to May 25, 2020 with the use of keyword ‘‘mosaic embryo transfer’’. We included studies according to participants, interventions, comparisons, outcomes and study design (PICOS) [27]. To be specific, included studies were case–control studies in which patients underwent IVF-PGT treatments, accepting either mosaic embryo transfer or euploid embryo transfer with trackable clinical outcomes (implantation/clinical pregnancy, pregnancy loss, ongoing pregnancy and/or live birth) between an MET group and a control group. Any missed information regarding the five key elements will be excluded, as well as conference abstracts, opinions, letters, views and reviews, surveys, case reports and committee opinions. Given that not all studies had live birth records at the time of publication, ongoing pregnancies and live births were analyzed together in some of the studies.

### 2.9. Statistical Analyses

Pearson chi-square test was performed to test the outcome difference between MET and control groups. Fisher’s exact test was used for sample size smaller than 30. Two-sided significance of <0.05 was regarded as statistically significant compared to the null hypothesis. Statistics for this study was performed by SPSS (IBM SPSS Statistics, version 25).

Meta-analysis was performed by Review Manager version 5.4 [28]. Publication bias was firstly evaluated by funnel plots. The heterogeneity of studies was assessed by I-squared test. When *p*-value of the heterogeneity test >0.1, the studies had sufficient similarities to be pooled together for analysis and a fixed model was performed. When the heterogeneity test had *p* < 0.1, a random effect model was applied with the outlier study subgrouped for comparison. Moreover, I^2^ > 50% was considered as moderate to high heterogeneity between studies and therefore was not eligible for inclusion of meta-analysis. Mantel–Haenszel (M–H) pooled risk ratios (RR) with a 95% confidence interval of implantation, miscarriage and ongoing pregnancy/live births were undertaken.

## 3. Results

Among the 137 mosaic embryos transferred, 55 embryos from 54 FET cycles were successfully implanted (IR: 40.1%, 55/137) with statistical difference (*p* < 0.001) when compared with the euploid control (IR 59%, 281/476), but not when compared with the non-PGT control (IR 45.7%, 382/835, *p* = 0.153). Moreover, embryos with mosaicism had a statistically lower chance of reaching clinical pregnancy in the MET group (CPR 40.6%, 54/133) than in the euploid control (CPR 59.1%, 264/447), but not in the non-PGT control (CPR 48.4%, 376/777). Thirty-six pregnancies (37 embryos) in the MET group led to ongoing pregnancies and live births, with an OPR of 27.1% (36/133), which was significantly lower (*p* < 0.001) than the OPR of 47.0% (210/447) in the euploid control. The OPR was also lower in the MET group than the non-PGT control (OPR 35.1%, 273/777), but with no statistical significance (*p* = 0.468). The miscarriages in the MET group occurred ranging from week 6 to week 17 of gestation, with the majority during the first trimester. The miscarriage rate was higher in the MET group (33.3%, 18/54) than in the euploid control (20.5%, 54/264, *p* = 0.05), but there was no statistical difference between the MET group and the non-PGT control (27.4%, 103/376, *p* = 0.652) (Table 2). In general, the pregnancy outcomes from METs were statistically worse than euploid transfers, but there was no significant difference when compared to the non-PGT controls.

### 3.1. Outcome Predictors of Mosaic Embryo Transfers

Embryos in the MET cohort had an average mosaic level of 37% ± 11% with a range of 20% to 69%. On the other hand, pregnancy outcomes were globally decreased in the <50% level of mosaicism when compared to euploid transfers. The number of embryos with a mosaic level of <40% that reached clinical pregnancy and ongoing/live birth (also when the mosaic level ≥40%) was significantly less than euploid ETs. No significant difference was found within the MET group when 40% and 50% were set as the cut-off level of mosaicism (Table 3).

Pregnancies ending in miscarriage in MET were significantly different between embryos carrying numerical (10 embryos) and segmental (8 embryos) chromosomal abnormalities (Fisher’s exact test, *p* = 0.04). However, no difference was observed in clinical pregnancies (20 numerical vs. 35 segmental, *p* = 0.79) and ongoing pregnancies/live births (10 numerical vs. 27 segmental, *p* = 0.23) (Table 4).

Pregnancy outcomes were compared between mosaic and euploid control embryos with different blastocyst morphological gradings that were available. Embryos with “good” morphology from the MET group (41.5%) had a statistically lower chance of reaching clinical pregnancy than those of the control group (61.7%, *p* = 0.021). Embryos with “fair” morphology consistently showed poorer outcome in the MET group (Table 5).

### 3.2. Systematic Review and Meta-Analysis

**Figure 1:**
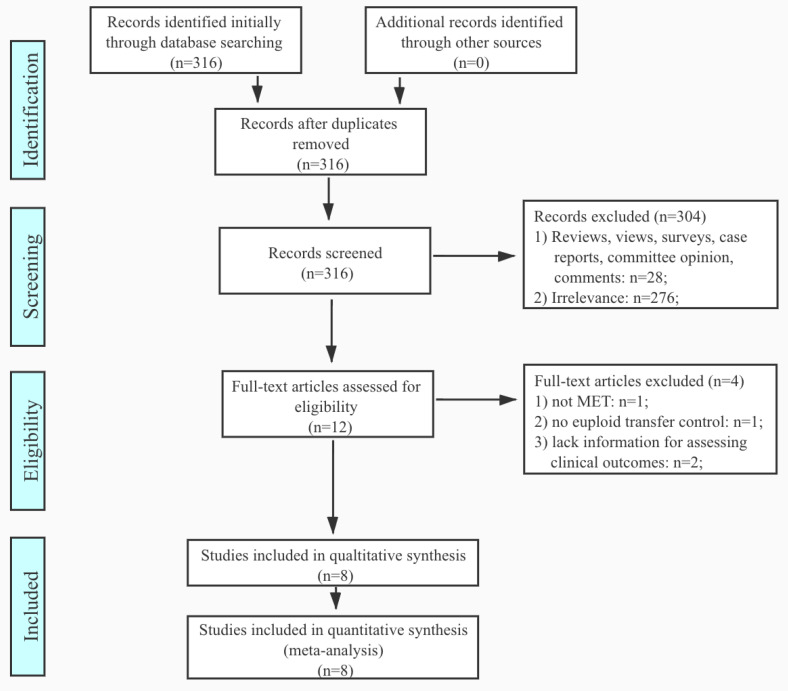
Preferred Reporting Items for Systematic Reviews and Meta-analyses (PRISMA) flow chart of literature search by the application of inclusion and exclusion criteria.

**Figure A1:**
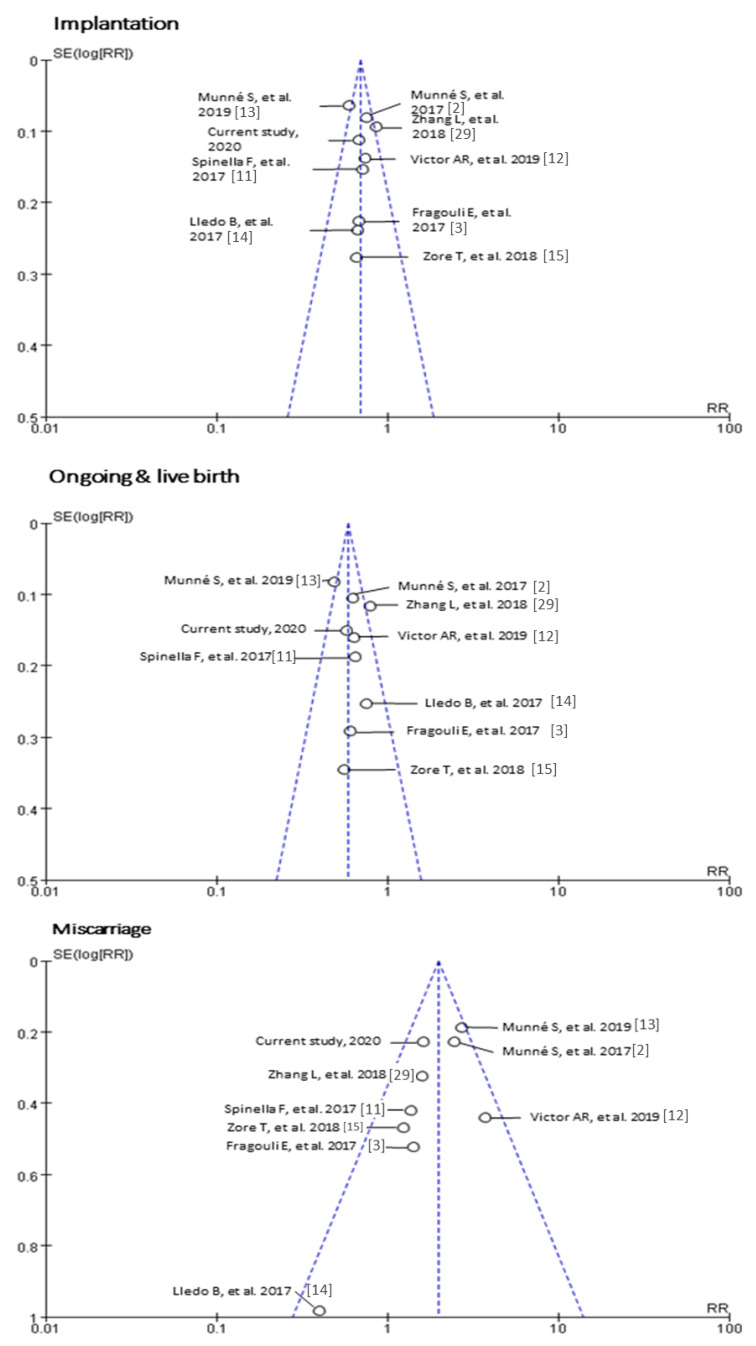
Funnel plot of the nine studies included for meta-analysis.

By searching the available literature on PubMed using “mosaic embryo transfer”, 316 results returned. There were 276 irrelevant publications, and 28 entries of views, reviews, surveys, case reports and committee opinions were excluded. Among the 12 eligible studies, one study was not focused on mosaic transfer, one report was without control, and two studies reported insufficient clinical pregnancy outcomes. After filtrations, eight studies were included for this meta-analysis (Figure 1). The eight studies uniformly performed PGT-A by NGS or CMA. All studies included for meta-analysis compared implantation/clinical pregnancies, miscarriage, ongoing pregnancies, and/or live births to euploid embryo transfers. As not all studies had live birth records at the time of publication, ongoing pregnancies and live births were analyzed together in some of the studies. Therefore, for the uniformity of study methodology, this meta-analysis combined analysis of ongoing pregnancies and live births. Heterogeneity analysis by funnel plot of the included studies can be found in Figure A1.

**Figure 2:**
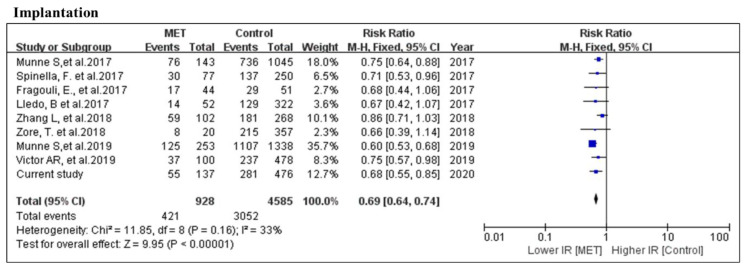
Meta-analysis of the comparison of pregnancy outcome between mosaic and euploid embryo transfers. Data from previous publications [2,3,11,12,13,14,15,29] and the current study. Comparison between MET and euploid ETs for the number of implanted embryos.

There were 421 mosaic (928 embryo population) and 3052 euploid (4585 embryo population) embryos transferred that resulted in implantation among the 9 studies that were included for meta-analysis of implantation rate. There was no statistical difference in overall heterogeneity. By a fixed model, the implantation rate was significantly lower in the MET group (RR = 0.69, 95% CI: 0.64–0.74, *p* < 0.001; nine studies; *I*^2^ = 33%, *p* = 0.16). None of the nine studies opposed this conclusion (Figure 2).

**Figure 3:**
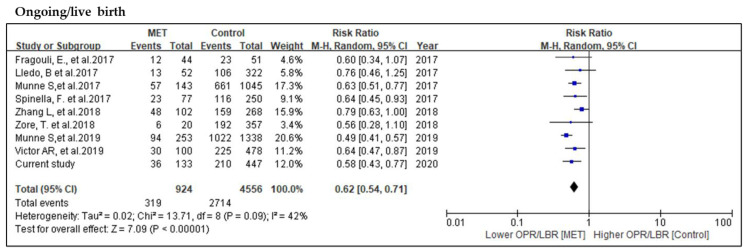
Meta-analysis of the comparison of ongoing pregnancies/live birth rates. Meta-analysis performed by the random effect model due to the fact that *p*-value of the heterogeneity test was less than 0.1.

Meta-analysis was conducted on nine MET studies for the comparison of ongoing/live birth rate. Among these 924 mosaic embryos transferred, 319 reached ongoing pregnancies/live births, as compared to 2714 from 4556 euploid embryos. A slight heterogeneity was observed; therefore, a random effect model was performed. Meta-analysis by the random effect model showed a significantly decreased possibility of reaching ongoing pregnancies/live births in MET when compared to the control cohort (RR = 0.62, 95% CI: 0.54–0.71, *p* < 0.001; nine studies; *I*^2^ = 42%, *p* = 0.09) (Figure 3).

**Figure 4:**
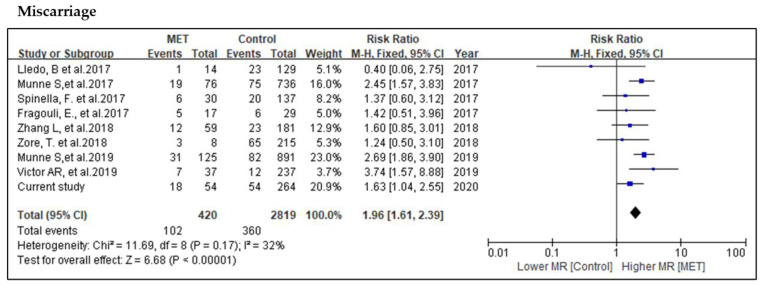
Meta-analysis of the comparison of miscarriages.

In the overall assessment of miscarriage rate, 102 mosaic (420 embryo population) and 360 euploid (2819 embryo population) embryos were reported as miscarriage. Given that the overall heterogeneity showed no difference with *p* = 0.17, this comparison was analyzed by a fixed model. The probability of miscarriage in MET is significantly higher than the control group (RR = 1.96, 95% CI: 1.61–2.39, *p* < 0.001; nine studies; *I*^2^ = 32%, *p* = 0.17) (Figure 4).

### 3.3. Perinatal Follow-up of MET Pregnancies

Twenty-five out of 36 live births (69.4%) among MET and 64 out of 109 live births (58.7%) in the control had birth weight records. No difference was found in regard to birth weight between babies from MET (3180 ± 505 g) and those in the control group (3047 ± 560 g, *p* = 0.325) (Table 1). Among the MET live births, four single embryo transfers (SETs) underwent non-invasive prenatal testing with no chromosomal abnormality detected. One SET underwent amniocentesis, with euploid results from both karyotyping and CMA. There were two pregnancy losses that proceeded with karyotyping on POG. One loss occurred at 8 weeks of gestation and another one beyond 20 weeks, both showing a euploid karyotype.

### 3.4. Postnatal Confirmation by Placental Multiple Biopsy

**Figure 5:**
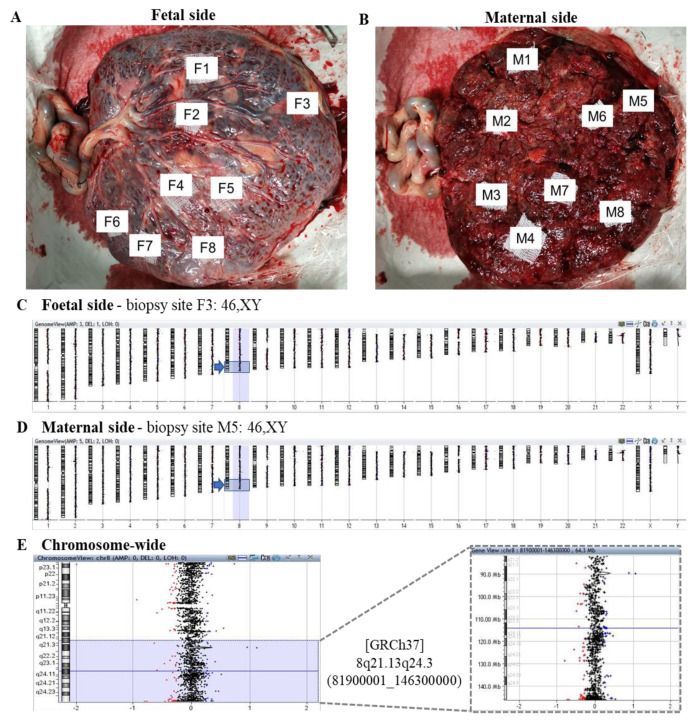
Postnatal CMA confirmation of one MET pregnancy by placental multiple biopsies. (**A**,**B**) Biopsies on eight sites from each side of the placenta. Male control as a reference for chromosomal microarray analysis, showing an example of absence of segmental chromosomal aneuploidy on (**C**) the fetal side view, (**D**) the maternal side genome view, (**E**) and the chromosome and regional view of [GRCh37] 8q21.13q24.3 (81900001_146300000), with no chromosomal aberration.

The MET case with negative amniocentesis had a full-term delivery and donated the placenta for research. The patient transferred a male embryo with 45% mosaic loss at [GRCh37] 8q21.13q24.3 (81900001_146300000), 64.4 Mb in size. By CMA on all 16 biopsies (Figure 5A,B), it was observed that 2 sites from the fetal side and one site from the maternal side had two copies of chromosome X, indicating maternal DNA contamination. All the other 13 biopsies showed a euploid result (46,XY) (Figure 5C–E).

## 4. Discussion

Our current multi-center study demonstrated the decreased viability of mosaic embryos and concluded with the same findings as all MET clinical studies by meta-analysis. With the previous proof-of-principle study [30] and mouse model [8], we proposed that the transfer of mosaic embryos could result in viable and genetically normal live births. Prior studies compared the pregnancy outcomes of METs to euploid ETs and had consensus on the poorer outcome by METs [2,3,11,12,13,14,15,29,30]. To expand the population of METs from the previous studies and to further investigate the genetic outcome of MET pregnancies, we conducted this multi-center prospective study, reaffirming the pregnancy outcomes of METs and reporting complete pregnancy outcomes with prenatal and POG genetic follow-up.

Different to the previous study using 50% as cut-off [11], we tested the effect of cut-offs at levels of mosaicism on both 40% and 50%. However, our cohort did not observe any significant difference between higher and lower mosaic level when the cut-off was at 40% or 50%. This may be due to limited size of mosaic embryos with levels greater than 40% and 50%. Nevertheless, our study provided meta-analysis data regarding METs. Pregnancies ended in miscarriage in MET were significantly higher in embryos carrying numerical compared to segmental chromosomal abnormalities. This was consistent with the study by Zore et al. who intentionally selected ETs with segmental mosaicism to fill the gap of current knowledge regarding the outcome of segmental copy number variants (CNVs). This study evaluated 20 embryos with segmental mosaicism that were transferred and concluded a reduced pregnancy outcome as compared to euploid ETs [15]. Victor et al. expanded this to compare the difference between single segmental mosaicism and other mosaicisms [12]. Taken together, our data suggested that the pregnancy outcomes of embryos with single segmental mosaicism had better prognosis, and this could be a selection criterion for MET.

The effectiveness of MET studies has been debatable on the detection accuracy of mosaicism and the aneuploid calling consistency between TE and inner cell mass (ICM). Capalbo et al. discussed the possibility of false positives in mosaic callings and suggested additional biopsies from the embryo reported, as mosaic embryos should be tested to confirm their true chromosomal constitution [31]. Victor et al. comprehensively addressed these doubts by performing cell line mix for detection accuracy and embryonic mosaicism confirmation by Fluorescence in situ hybridization (FISH) [12]. By testing 30 blastocysts, their study suggested both TE and ICM of mosaic embryos were at a significantly higher rate of mitosis and apoptosis than euploidies but were at a similar rate to aneuploidies. Moreover, an additional biopsy on mosaic embryos showed reciprocal copy number variation in TE but euploidy in ICM, providing solid evidence of chromosomal nondisjunction. In a study of multiple blastocyst biopsy for the consistency of TE and ICM, Victor et al. reported a consistency of 96.8% when the aneuploidy involved one or more numerical chromosomes [32]. Even though the rate of embryonic mosaicism has been estimated to account for 3% to 86% in blastocysts [5,6], the true fetal mosaic (TFM) rate is only 0.2% in prenatal specimens and 0.1% in products of gestations [23,33,34]. Except potential technical issues of PGT-A, this large variation of occurrence between the preimplantation and prenatal stage indicated that the genetic aberrations detected in the preimplantation stage do not all developed into later stages. This can be explained in two aspects: Firstly, aneuploidy rescue might occur during embryonic development, in-line with the aneuploidy cell line depletion theory supported by a chimeric mouse study [8]. Secondly, the reproducibility of mosaicism on re-biopsies from trophectoderms was only 41–58% [1,35], demonstrating that single biopsy employed in PGT-A might not precisely represent the true genetic constitution and level of mosaicism. Therefore, current PGT-A reporting and embryo transfer criteria have reduced the number of embryos that are potentially viable by overestimating the detrimental effect of mosaic embryos. Further investigation into products of gestation, prenatal specimens or placental tissue of MET pregnancies by a sequencing approach is warranted to reveal low-level mosaicism and copy number variants [36,37].

Although mouse model [8] and clinical studies supported the live-born possibility of low-level mosaic embryos, none specified the differential risk of each chromosome on later development. Grati et al. established a practical scoring system based on prenatal data to support the decision-making in PGT [23]. True embryonic/fetal mosaicism of sex chromosomes is viable and therefore scored as high risk due to the possibility of leading to abnormal live born. Thus, special awareness is needed in mosaicism of sex chromosomes due to compromised sequencing quality at repetitive sequence regions and the viability of embryos carrying sex chromosomal aneuploidies. In our study cohort, sex chromosome mosaicism was detected in 10 embryos, resulting in 7 women with no pregnancy, 2 biochemical pregnancies and 1 live birth. The PGT-A result of this live born was low-level (<30%) mosaic XY/Y and mosaic trisomy 14 (<30%). The baby’s peripheral blood was retrieved and had a normal karyotype (46, XY). The likely explanation for this case and our longitudinal study is aneuploid cell depletion. However, a recent case report showed 2% mosaic monosomy 2 in amniotic fluid and peripheral blood of the baby developed from an embryo with 35% monosomy 2, raising further consideration of the developmental potential of aneuploid cells [38].

## 5. Conclusions

This multicenter prospective study in Asia has fully followed up the pregnancy outcomes after mosaic embryo transfer to the perinatal stage. Meta-analysis confirmed the consistently lower pregnancy outcome of MET compared to euploid transfers. Nevertheless, the viable and karyotypically normal live births by MET suggest it as an alternative option for patients with no euploid embryo acquired from PGT-A cycles. However, this option could only be provided to patients under the condition of comprehensive genetic counselling and recommendation to follow-up with prenatal diagnosis. A larger-scale randomized controlled trial is necessary to further evaluate specific numerical and segmental chromosomal effects of mosaic embryo transfer. Even though there is no evidence on confined placental mosaicism due to MET, more data on multiple placental biopsies from MET pregnancies are warranted for further investigation of mosaic embryo development.

## Figures and Tables

**Table 1:** Clinical information of studied cohorts.

	MET	Euploid Control	Non-PGT Control
**Age**	31.8 ± 6.4	34.5 ± 5.8	36.9 ± 3.3
**Embryos**	137	476	835
**ETs**	133	447	777
Single ET	129 (97%)	418 (94%)	719
Double ET	4 (3%)	29 (6%)	58
**Clinical Indications**			
AMA	56 (41%)	276 (58%)	/
RM *	4 (3%)	32 (7%)	/
RIF *	4 (3%)	17 (4%)	/
Translocation Carriers *	0	6 (1%)	/
Male factors *	0	3 (1%)	/
PGT-M *	2 (2%)	29 (6%)	/
Previous Abnormal Pregnancy *	6 (5%)	18 (4%)	/
Aneuploidy Screening *	54 (43%)	79 (17%)	/
**Morphology**			
Good	41 (30%)	141 (44%)	/
Fair	89 (65%)	160 (50%)	/
Poor	7 (5%)	19 (6%)	/
**Birth Weight (g)**	3180 ± 505	3047 ± 560	/

* these indications were counted mutually exclusive to AMA; i.e., if AMA co-existed with RM, the clinical indication was classified as AMA. AMA = advanced maternal age; RM = recurrent miscarriage; RIF = repeated implantation failure; MET = mosaic embryo transfer; non-PGT = non-preimplantation genetic testing; ETs = embryo transfers; PGT-M = preimplantation genetic testing for monogenic disorders.

**Table 2:** Pregnancy outcome variation between groups.

	Clinical Pregnancy	Ongoing Pregnancy/Live Birth	Miscarriage
**MET**	40.6% ^a^	27.1% ^a^	33.3%
**Euploid Control**	59.1%	47.0%	20.5%
**Non-PGT** **Control**	48.4% ^b^	35.1% ^b^	27.4% ^b^

^a^, *p* < 0.05 between MET and euploid control; ^b^, *p* < 0.05 between euploid and non-PGT control.

**Table 3:** Reproductive outcome of different levels of mosaicism compared to euploid embryo transfers.

	Clinical Pregnancy			Ongoing Pregnancy/Live Birth			Miscarriage		
	No. of Embryos	*p* *	*p* **	No. of Embryos	*p* *	*p* **	No. of Embryos	*p* *	*p* **
**Euploid**	281			223			58		
**Mosaic level**									
**<40%**	30	0.10	<0.001	21	0.38	<0.001	9	0.64	0.24
**≥40%**	25		0.17	16		0.04	9		0.08
**<50%**	47	0.27	<0.001	30	0.07	<0.001	17	0.19	0.02
**≥50%**	8		0.66	7		0.99	1		1 ***

* *p*-value were MET is compared within the MET group; ** *p*-value were MET is compared to euploid; *** chi-square test by Fisher’s exact test.

**Table 4:** Outcome comparison according to classification by the size of the largest variant.

Classification by Size of Mosaic Variant *	Clinical Pregnancy		Ongoing Pregnancy/Live Birth		Miscarriage	
	MET	*p*-Value	MET	*p*-Value	MET	*p*-Value
Numerical	20	0.79	10	0.23	10	0.04
Segmental	35		27		8	

* Mosaic numerical or segmental variants denoting the predominant variant in size detected per embryo. Neither chromosome nor mosaic level were verified.

**Table 5:** Outcome comparison by morphological grading.

Morphological Grading	Clinical Pregnancy			Ongoing Pregnancy/Live Birth			Miscarriage		
	MET	Control	*p*-Value	MET	Control	*p*-Value	MET	Control	*p*-Value
**Good**	17 (41.5%)	87 (61.7%)	0.021	16 (39.0%)	70 (49.6%)	0.23	1 (5.9%)	17 (19.5%)	0.173
**Fair**	35 (39.3%)	97 (60.6%)	<0.05	18 (20.2%)	74 (46.3%)	<0.005	17 (48.6%)	23 (23.7%)	0.006
**Poor**	3 (42.9%)	11 (57.9%)	0.67	3 (42.9%)	8 (42.1%)	1.00	0 (0%)	3 (27.3%)	1.00
**Within group difference (*p*-value)**	0.96	0.94		0.05	0.75		< 0.05	0.72	

Good including “AA/AB”; fair including “BA/BB/BC”; and poor including “CB/CC”.
